# Hydrogel-Embedded Polydimethylsiloxane Contact Lens for Ocular Drug Delivery

**DOI:** 10.1021/acsabm.4c00975

**Published:** 2024-10-19

**Authors:** Aravind Manjeri, Sajan Daniel George

**Affiliations:** † Department of Atomic and Molecular Physics, Manipal Academy of Higher Education, Manipal 576104, India; ‡ Centre for Applied Nanosciences (CAN), Manipal Academy of Higher Education, Manipal 576104, India

**Keywords:** microfluidic, contact lens, drug delivery, on-demand drug release, pH-responsive

## Abstract

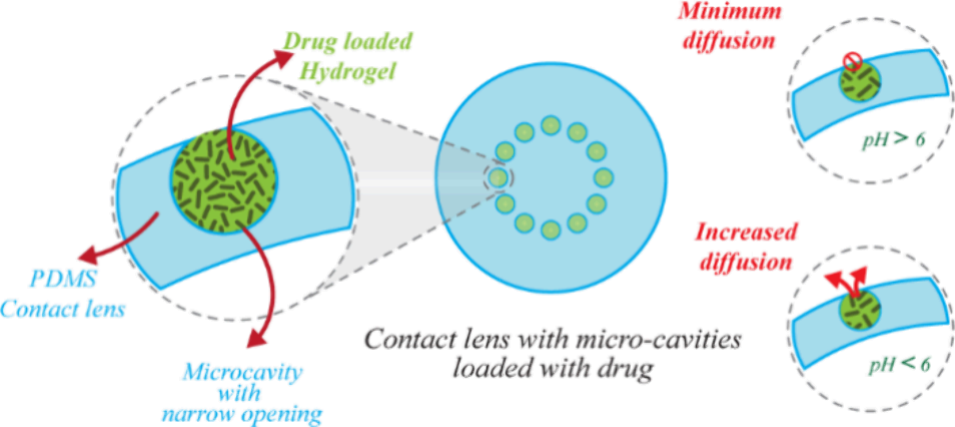

Topical administration is the commonly preferred method of administering ophthalmic formulations, with the majority of available medications in the form of eye drops or ointments. However, the topical application of ophthalmological medications has less bioavailability and a short residence time because of the physiological and anatomical constraints of the eye, making efficient ophthalmic drug delivery a challenging task. Microfluidic contact lenses have the advantage of delivering drugs into the eye in a controlled and on-demand manner. Here, we showcase the use of hydrogel-embedded microcavities on PDMS-based contact lenses for ocular drug delivery applications. The fabrication technique adopted here is the spontaneous formation of the spherical cavity by hydrogel monomer droplet, followed by the simultaneous thermal curing of hydrogel and PDMS, creating a spherical cavity as small as 150 μm. The spherical cavity is embedded with pH-responsive hydrogel for on-demand drug delivery. The drug loaded in the hydrogel matrix is released into the ocular environment by diffusion. The spherical cavity with a narrow opening restricts the diffusion to a minimum under normal ocular pH conditions(pH > 6). When the ocular pH reduces (pH < 6), the pH-responsive hydrogel inside the spherical cavity deswell and accelerates the drug release.

## Introduction

Microfluidic contact lenses have attracted particular interest in the past few years due to their potential of allowing on-eye, real-time tear fluid processing. Ocular diagnosis by evaluating various tear components, such as chloride ions, nitrite ions, urea, pH, and proteins using microfluidic architecture, has proven to provide crucial insights into ocular health.^1−3^ Developments in microfluidic contact lens research have led to significant improvements in monitoring intraocular pressure (IOP), the indicator of glaucoma. Microchannel-embedded contact lenses have shown promising results in continuous IOP monitoring without any electrical components.^4−6^ One potential advantage of microfluidic contact lenses is the ability to deliver drugs into the eye in a controlled and on-demand manner. Topical instillation is the most commonly used noninvasive method of administering drugs to treat eye diseases. Conventional dosage forms, such as eye drops, make up 90% of the ophthalmic formulations available on the market. This is likely due to the ease of administration and patient compliance.^7,8^ Due to anatomical and physiological factors, such as tear turnover, nasolacrimal drainage, reflex blinking, and ocular static and dynamic barriers, topically instilled medications have low bioavailability and a short residence time of less than 2 min. Consequently, less than 5% of the applied dose is effectively available, as these factors pose challenges for the medication to permeate deeper into the eye tissues.^9,10^ To overcome the limitations of traditional ophthalmic medications, researchers are putting effort into creating innovative drug delivery approaches with extended retention times and high bioavailability. Drug-loaded contact lenses are emerging as new drug delivery tools that utilize various techniques to incorporate ophthalmic drugs into contact lenses. These lenses have garnered significant attention due to their ability to address issues related to inaccurately administering eye drops. Additionally, they can provide continuous drug release into the tear film, prolong drug retention time on the ocular surface, and enhance bioavailability.^8^ Though drug-loaded contact lenses show great promise, there are still challenges to overcome to make them ideal candidates for routine usage. For example, drug-loading can alter the critical properties of contact lenses, leading to low water content, reduced oxygen and ion permeability, impaired transparency, and undesirable drug release that may occur during postproduction monomer extraction.^11,12^ Nevertheless, incorporating ophthalmologic drugs into the microfluidic components of contact lenses enables controlled, on-demand drug release. Drug release from the microreservoirs of contact lenses in response to a trigger is an effective approach for efficient on-demand ocular drug delivery.

Studies have reported using external as well as internal stimuli to release drugs from the microreservoirs of a contact lens. A magnetic micropump-embedded contact lens where the drug release is based on a magnetically actuated check valve connected to a drug-loaded chamber is an example of an externally controllable drug delivery mechanism.^13^ However, the drug release from internal ocular stimuli could be more advantageous, such as the IOP-triggered drug release and blinking pressure-triggered drug release.^14,15^ The fabrication techniques for microfluidic contact lenses can be complex and often involve multiple steps, making mass production and scalability difficult. The conventional microfabrication process to obtain microfluidics devices is a planar process, and employing it directly to fabricate 3D spherical devices like contact lenses is challenging.^16^ The reported approaches for fabricating microfluidic contact lenses include thermoforming, where a thermoplastic like polyethylene terephthalate (PET) bonded with the microfluidic elements replicated a soft polymer such as polydimethylsiloxane (PDMS) and thermoformed to the contact lens curvature.^4,17^ Researchers have also used a faster approach by inscribing microfluidic elements directly onto the contact lens surface using a laser, compared to the previous method.^18,19^ Though microfluidic contact lens drug delivery offers several advantages, the fabrication of microfluidic contact lenses involves expensive and complex techniques, creating a demand for a simpler approach to forming micro reservoirs on contact lenses.

Here, we demonstrate a method to fabricate spherical microcavities and hydrogel-embedded microcavities with narrow openings on PDMS by spontaneous and self-formation with high-surface tension liquid droplets. The droplet template method offers a significant advantage over conventional techniques, which are typically limited to creating planar substrates. This method enables the easy fabrication of spherical cavities with narrow openings, a shape that is challenging to achieve using traditional approaches. The microcavity contains hydrophilic water-absorbing hydrogels that can hold various drugs. These drugs can be released into the eye through a narrow opening; also, the hydrophobic PDMS ensures that the hydrogel remains confined within the cavity. The single-step fabrication of stimuli-responsive hydrogel-embedded microcavities and drug release is demonstrated. The pH-responsive hydrogel that was fabricated has shown a drastic reduction in swelling when the pH is reduced from the normal ocular pH. The minimum diffusion at the swollen state and the drug release by deswelling are exploited in the study for on-demand drug release.

## Experimental Section

### Materials

Polydimethylsiloxane (Sylgard 184, Dow Corning), 2-hydroxyethyl methacrylate (96.6%, extra pure, HEMA, Loba), ethylene glycol dimethacrylate (98%, EGDMA, Sigma-Aldrich), azobis(isobutyronitrile) (98%, AIBN, Loba), acrylic acid (99%, Molychem), glycerol anhydrous (Merck), ethanol (100%, Hayman), acridine orange (Practical grade, Himedia), and rhodamine 6G (Sigma-Aldrich) were employed.

### Fabrication of Microcavity in PDMS

The liquid PDMS is prepared by mixing the base (Sylgard 184, silicone elastomer base) and cross-linking agent (Sylgard 184, silicone elastomer curing agent) thoroughly in a ratio of 10:1 and desiccated to remove the air bubbles. The liquid PDMS is transferred to a Petri dish for droplet dispensing. A syringe filled with the test liquid, either glycerol for fabricating empty cavities or a hydrogel monomer for hydrogel-embedded cavities, is loaded into a syringe pump (Pump 11 Elite, Harvard Apparatus). This syringe pump is mounted on a programmable XYZ motorized translation stage (Holmarc, India). The system is programmed to gradually lower the syringe, allowing a droplet to be carefully placed onto the liquid PDMS surface (Figure S1). The PDMS is cured at a temperature of 90 °C for 1 h, followed by cleaning with ethanol to remove the remaining test liquid. The cavity radius and orifice size are measured using microscopic images, and the data plotted represent the average of five separate readings.

### Fabrication of Hydrogel-Embedded Microcavity in PDMS

The hydrogel monomer solution is prepared by mixing monomer HEMA with 0.1 wt % of the cross-linker EGDMA and 0.1 wt % of the initiator AIBN in solvent glycerol. The mixture is thoroughly stirred before being loaded into the syringe. The solvent glycerol percentage is changed from 0 to 75% to find the optimum solvent concentration that allows the swollen hydrogel radius to match with the cavity radius. The pH-responsive hydrogels were prepared by adding 10% acrylic acid to the previously optimized monomer solution. One mL of the monomer solution was loaded into the syringe, and droplets were dispensed to liquid PDMS. Once the hydrogel monomer solution is dispensed and droplets sink into the liquid PDMS, both liquid PDMS and hydrogel monomer are thermally cured simultaneously at a temperature of 90 °C for 1 h. The hydrogel beads are fabricated by dispensing monomer droplets onto a PDMS base, followed by heating at 90 °C for 1 h. Since no curing agent is added, PDMS remains uncured. The hydrogel beads are then extracted from the base and thoroughly cleaned by using ultrasonication with ethanol and water. The clean and dry hydrogels are infused with fluorescent dye by immersing the substrates in the dye solution overnight. The swelling ratio of the hydrogels is calculated by the following formula, and the data plotted are the average of three measurements. The hydrogels are first weighed in the dry state; the dry weight (*W*_d_) is obtained, and the wet weight (*W*_w_) is measured after complete swelling, and the swelling ratio is given by
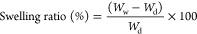


### Contact Angle Measurements

The surface wetting properties were examined by using a commercial contact angle instrument (Holmarc, India). Contact angles were measured by using a 5 μL drop on a flat surface. Hydrogel and PDMS plane substrates were fabricated, and contact angle data were collected from at least three different positions on each sample.

## Result and Discussion

### Spherical Cavity Formation in PDMS

**Figure 1:**
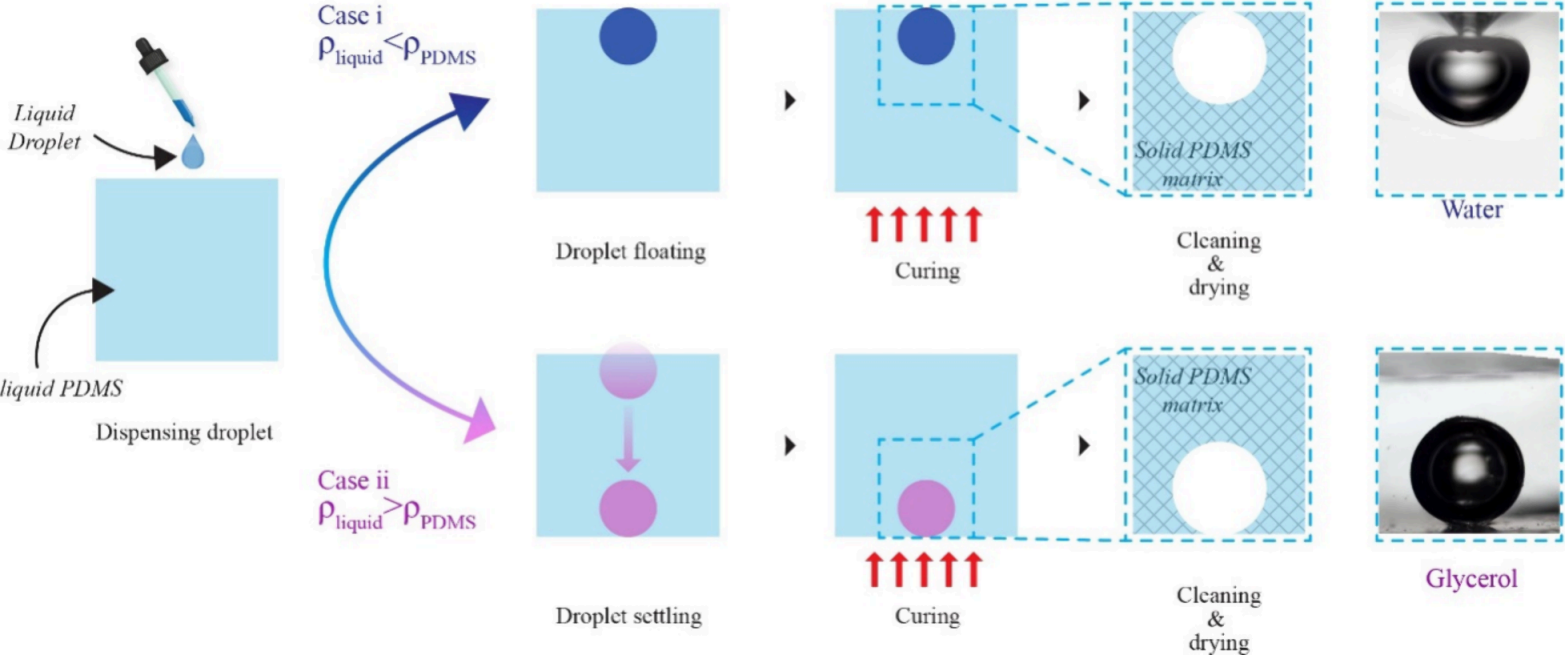
Schematic of the process of spontaneous formation of a spherical cavity by a liquid droplet on liquid PDMS.

The spontaneous self-formation of spherical cavities with high-surface tension liquid droplets in liquid polydimethylsiloxane (PDMS) is used here for the fabrication of microcavities with narrow openings. When a high-surface tension liquid droplet is placed on the surface of the liquid PDMS, the droplet is immediately surrounded by the liquid PDMS, and the space occupied by the droplet makes room for the cavity as the PDMS is cured.^20^ PDMS is used for the study as it is a transparent, flexible, biocompatible material and has been explored in a wide range of bioapplications. It has high oxygen permeability, making it advantageous for making contact lenses. PDMS is extensively used in the literature for the fabrication of contact lenses, especially for smart contact lenses with applications in drug delivery, sensing, and so forth.^14,21−24^ Additionally, contact lenses obtained with hydrophilic materials like poly(2-hydroxyethyl methacrylate) as the core polymer are unsuitable to be used as microfluidic platforms since the liquid spreads all over the contact lens. In contrast, hydrophobic materials such as PDMS are suitable for confining the contents of microfluidic elements. Here, once the droplet is dispensed to liquid PDMS, the surface tension of the droplet determines the final shape of the droplet inside the liquid PDMS. Since the study is making use of only high-surface tension liquids such as water, glycerol, and so forth. the droplet retains a spherical shape inside liquid PDMS. The next crucial factor is the liquid’s density, which determines the droplet’s position within the PDMS liquid; here, two cases are examined where the density of the liquid is above and below the PDMS density, as shown in Figure 1. In the first case, where the liquid is lighter than the surrounding liquid PDMS (ρ_liquid_ < ρ_PDMS_, ρ_PDMS_ = 1.028 g/cm^3^), the case is examined with water as the test liquid (ρ_water_ = 1 g/cm^3^).^25^ The lighter test liquid droplet is positioned near the top surface of the PDMS. On curing the PDMS, the space occupied by the liquid makes room for the cavity; the deviation from a completely spherical shape in this case is because of the evaporation of the test liquid. The opening near the liquid PDMS surface becomes the opening of the cavity. In the second case (ρ_liquid_ > ρ_PDMS_), where the test liquid is denser than the liquid PDMS, the droplet completely sinks to the bottom of the liquid PDMS. Here, glycerol (ρ_glycerol_ = 1.26 g/cm^3^) is used to examine the case, and as expected, the droplet sinks to the bottom of liquid PDMS, and curing the space occupied by the droplet leaves room for the cavity. Since the glycerol droplet is enclosed in the liquid PDMS, the cavity formed is spherical, and unlike the previous case, evaporation of the test liquid is not affected, and this offers better control and reproducibility over the cavity formation.

**Figure 2:**
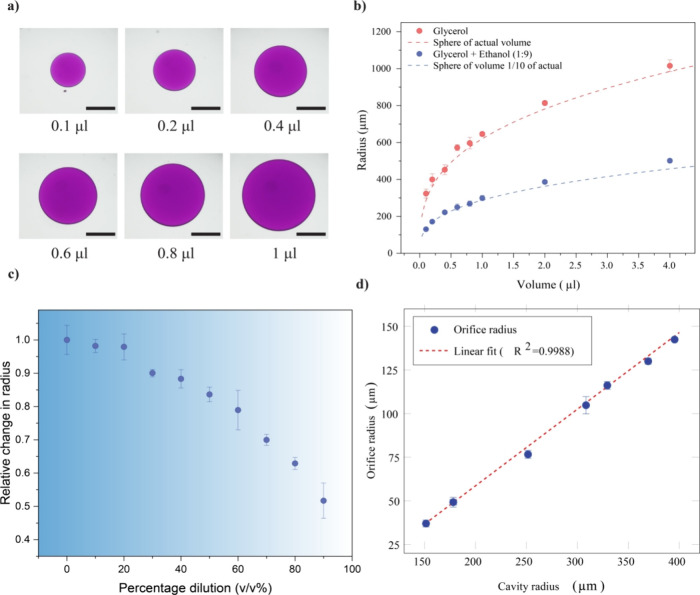
(a) Different volumes of glycerol dispensed on liquid PDMS (Scale bar = 400 μm), (b) effect of droplet volume on the cavity radius without dilution and with 90% dilution, (c) effect of dilution of the change in the cavity radius, and (d) change in orifice size with cavity size.

The cavity formation by a dense high-surface tension liquid such as glycerol on PDMS is a facile method for creating a spherical cavity. The dimension of the cavity formed is proportional to the volume of the test liquid droplet dispensed, therefore allowing the fabrication of cavities of various sizes by changing the droplet volume (Figures 2a and S2). Due to the spherical shape of the cavity, the dispensed volume is proportional to the cube of its radius; measurements of the radius from microscopic images also confirm this relationship (Figure 2b). The smallest cavity radius achievable using this setup was nearly 324 μm, corresponding to a droplet volume of 100 nL. To fabricate microcavities, the droplet volume dispensed also must be smaller, and dispensing small droplets of volume will require expensive, complex equipment. Here, we use a simple method to further reduce the size of the cavity by introducing a volatile component, such as ethanol, to the test liquid. The diluted test liquid is loaded into the syringe, and droplets are dispensed, where the volume is set to the minimum volume that the developed experimental setup can accurately dispense. Once the droplet is dispensed to the surface of the liquid PDMS, the volatile component in the droplet evaporates almost immediately, leaving behind a smaller amount of test liquid, in effect reducing the volume of the dispensed droplet. The volume of the test liquid remaining on the liquid PDMS is determined by the extent of dilution, and the effect of dilution on the cavity formation is studied by changing the percentage of dilution from 0 to 90% (Figures 2c and S3). The results showed that the dilution drastically reduces the cavity size; for a 90% diluted test liquid, the cavity radius is reduced by a factor of nearly 51%, which matches with the theoretical expectation of 46.4%. For a 90% diluted test liquid, the actual amount of the test liquid present is the remaining 10%, reducing the effective radius. The dilution method allows us to push the limit of the smallest cavity that can be fabricated, which can help in creating microcavities for miniature systems. As explained earlier, the denser test liquid sinks to the very bottom of the container of liquid PDMS; upon curing and removing the solid PDMS from the container, the point of contact of droplets leads to the fabrication of an opening to the cavity. The opening formed on the cavity is much smaller compared to the diameter of the cavity. Also, the smaller orifice of the spherical cavity is one of the unique characteristics of the structures, which can be challenging and labor-intensive on other fabrication approaches. The orifice size depends on the size of the cavity itself, and from the experiments, a linear relation between them is observed (Figures 2d and S4). The experimental setup is designed to fabricate an array of these microcavities in different sizes and shapes with the aid of an XYZ motorized programmable translation stage with the syringe pump mounted on it. In the case of a continuous fabrication approach, the syringe pump can offer a steady flow of the test liquid, which can be programmed to place different shapes and sizes. Here, the size of the microcavity depends on a few more parameters such as the translational stage speed and the flow rate, which control the volume of droplets dispensed at a particular spot (Figure S5). Tuning these parameters along with the volume and dilution allows one to obtain the desired cavity size.

**Figure 3:**
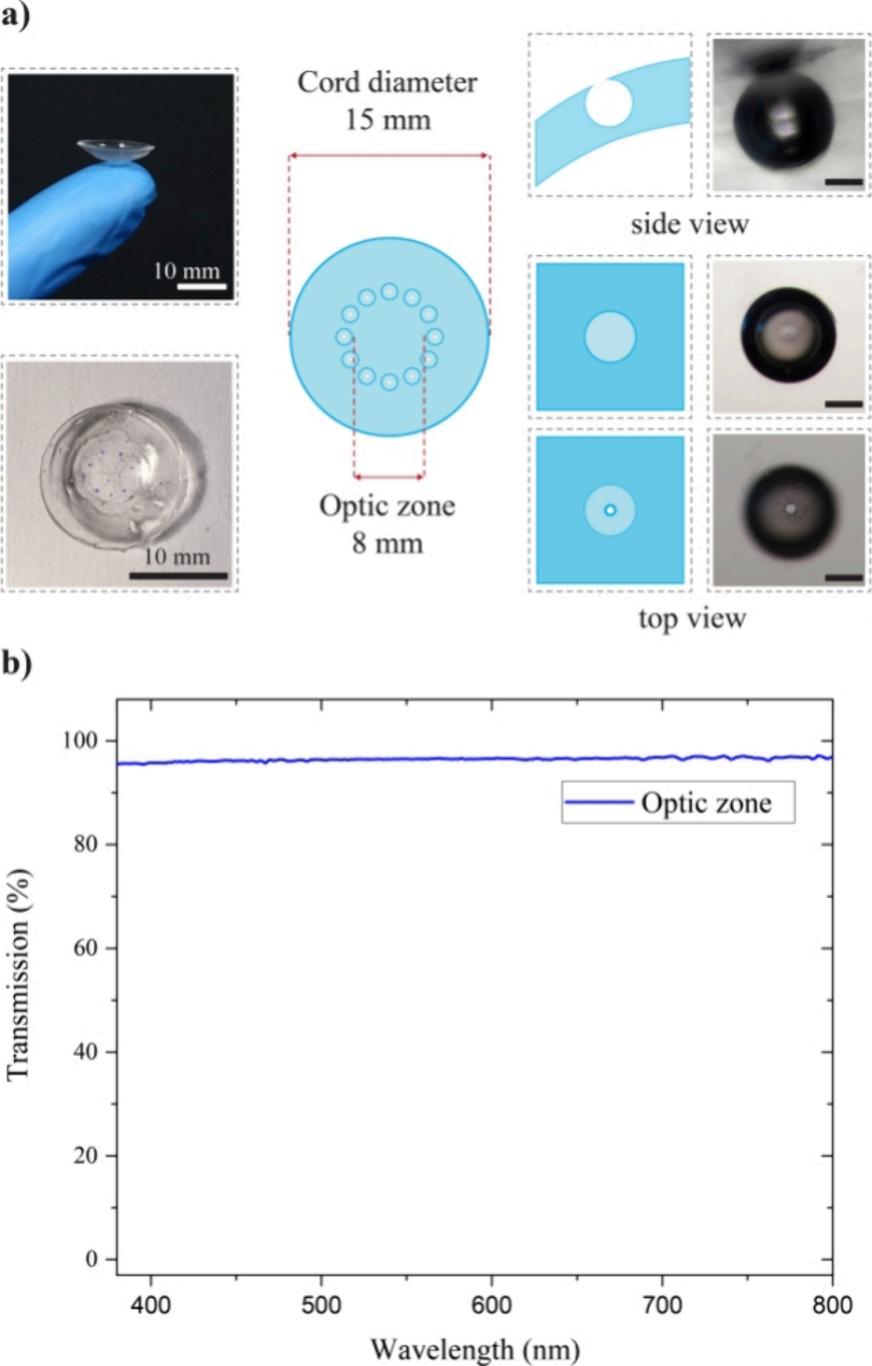
(a) Optical as well as microscopic images of the microcavity-embedded contact lens (Scale bar = 50 μm) and (b) transmission profile of the cavity-free optic zone.

The microcavities were fabricated on PDMS-based contact lenses; the fabrication approach integrated the mold casting method with the droplet dispensing setup (Figure S6). The cavity size control by the dilution method and flow rate control, as discussed previously, helps in fabricating smaller cavities. Especially in the case of contact lenses, having a larger cavity would naturally increase the contact lens thickness, which can reduce user comfort and adversely affect the total wearing experience. Here, nearly 150 μm diameter microcavities are fabricated on PDMS-based contact lenses, as shown in Figure 3a. During the fabrication process, the cavities are placed outside the optical zone (diameter 8 mm) such that it does not interfere with the vision, Also, the transmission profile of the cavity-free areas shows good optical transparency, as evident from the UV–vis transmission spectra (UV–vis spectrometer, Jasco) (Figure 3b).

### Hydrogel-Embedded PDMS Cavity

**Figure 4:**
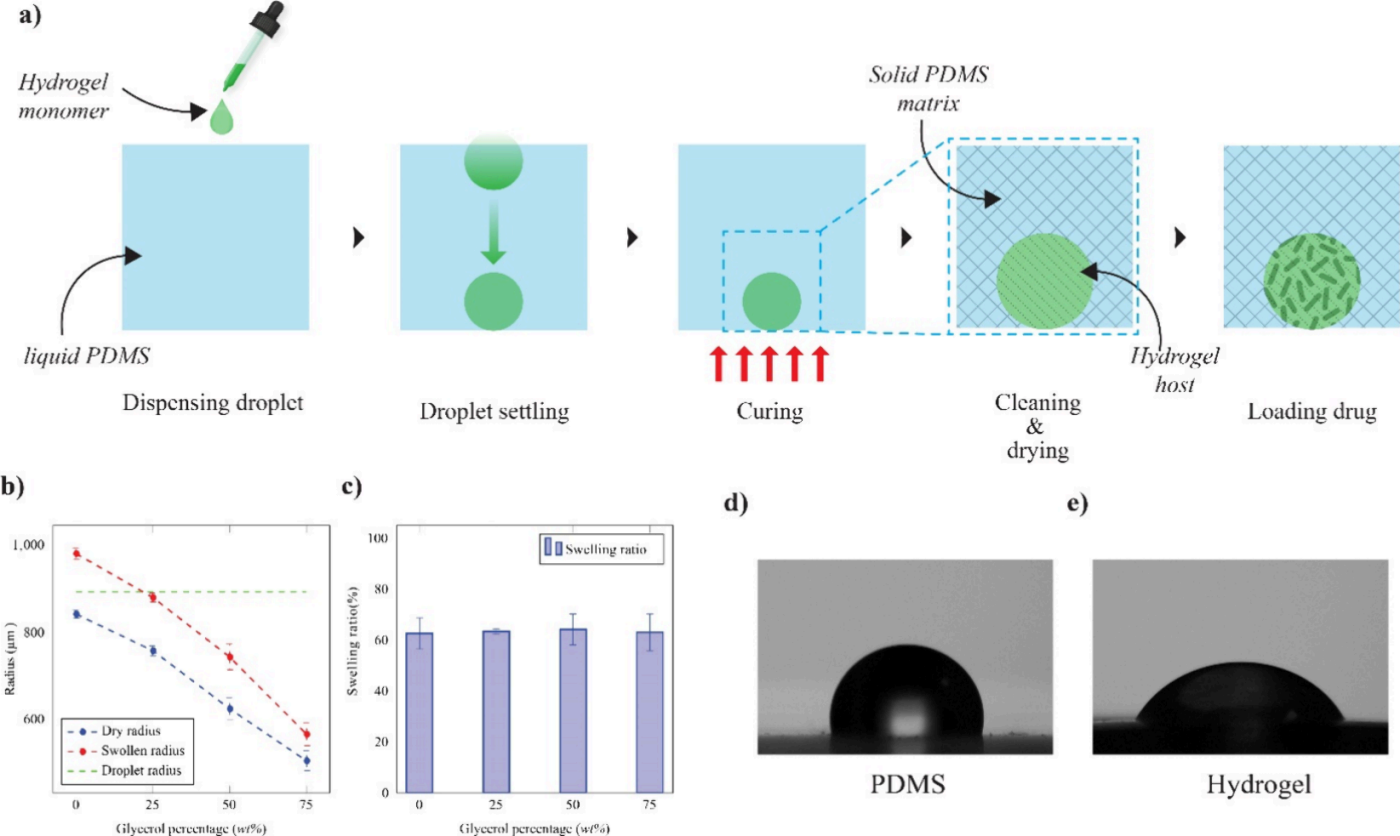
(a) Schematic of fabrication of hydrogel-embedded PDMS substrates, changes in the (b) radius and (c) swelling ratio of HEMA-hydrogel with changing solvent concentration, and the sessile drop images on (d) PDMS and (e) poly-HEMA surfaces.

Hydrogels are a class of polymers that can imbibe a large amount of water in their three-dimensional network structure. Environmentally sensitive hydrogels can serve a wide variety of applications because of their ability to respond to environmental changes, typically by exhibiting changes in volume. Traditional stimuli that cause hydrogel response are pH, temperature, and ionic strength.^26^ Because of such a wide variety of response triggers, hydrogels can be incorporated into sensors or actuators or can be utilized in controlled drug delivery systems.^27^ Here, we adopt a strategy to embed a hydrogel into the PDMS polymer. Replacing the test liquid used for fabricating the microcavity with the hydrogel monomer results in the spontaneous and simultaneous formation of a hydrogel embedded in the PDMS cavity. Choosing a thermal initiator such as AIBN for the polymerization of the hydrogel allows the simultaneous and rapid fabrication of the hydrogel along with the thermal curing of PDMS (Figure 4a). The test liquid used results in the formation of a spherical cavity equivalent to its volume, as observed in previous cases. Once the hydrogels are polymerized inside the cavity, it is cleaned and dried, and upon rehydration, the hydrogel beads swell inside the microcavity. The solvent concentration is carefully optimized to achieve a swelling ratio where the beads expand to a radius that matches the cavity’s radius. The hydrogel beads were fabricated with different solvent percentages, and the swelling ratio was evaluated. The study revealed that the optimized level of nearly 25% of the solvent is suitable for matching the hydrated size of the hydrogel with the cavity size (Figure 4b). Increased levels of solvent result in smaller swollen hydrogels, which can result in vacant space inside the cavity, and the hydrogel can swell and bulge above the cavity at a lower solvent concentration (Figures 4b and S7). Also, the fabricated hydrogels have a swelling capacity of nearly 60%, as shown in Figure 4c. The optimized solvent level is examined for various droplet volumes, resulting in expanded hydrogels that match the respective cavity size and exhibit the same swelling ratio (Figures S8 and S9). The surface wettability of the hydrogel and PDMS is investigated; the contact angle measurements establish the hydrophilic nature of HEMA-based hydrogels with a contact angle 58 ± 2°, and the PDMS surface is hydrophobic with a 102 ± 2° contact angle (Figure 4d,e).

**Figure 5:**
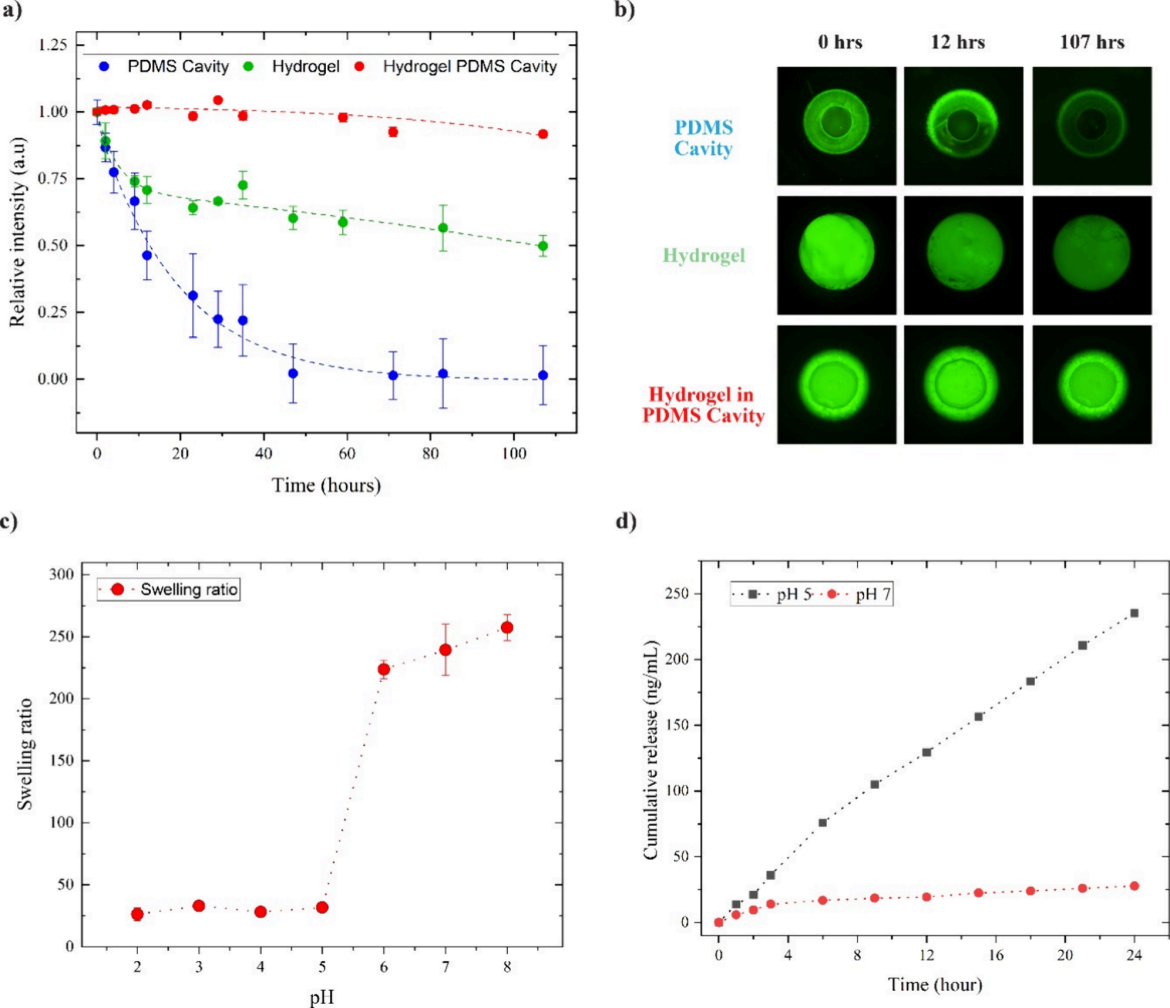
(a) Plot of fluorescent intensity changes from various substrates over time, (b) corresponding fluorescent images of different substrates at different points of time, (c) pH-responsive swelling of the hydrogel with changing solvent pH, and (d) dye release profile above and below the ocular pH (average of three data points and error bars are equal in size to the data points).

The ability of hydrogels to imbibe a large amount of water in their three-dimensional network structure makes them an ideal candidate for storing and releasing drugs to the surroundings. Though there have been studies on using drug-soaked contact lenses as a drug delivery platform, they suffer from different drawbacks since drug-loading may change critical properties of contact lenses such as poor mechanical strength, impaired transparency, and decreased oxygen permeability.^11^ Here, the drug-loaded hydrogel inside the PDMS-based spherical cavity can offer an alternative drug delivery strategy without compromising the optical property as well as the oxygen permeability. The drug loaded in the hydrogel can diffuse into the surroundings by diffusion through the small orifice. A fluorescent study is designed to investigate the diffusion behavior of components within the hydrogel using a fluorescent dye as a surrogate for the drug. Changes in the fluorescence intensity will provide insights into the diffusion process. The hydrogel host was soaked with a fluorescent dye (acridine orange) and immersed in a tear supplement; the fluorescent image was recorded at different intervals of time (fluorescence microscope, Eclipse Ni, Nikon), and the reduction of intensity is indicative of the diffusion. The PDMS cavity, hydrogel beads, and hydrogel inside PDMS cavity substrates were used for the comparative study, the fluorescent images obtained were analyzed with ImageJ software, and the intensity change over time was plotted to obtain the diffusion profile (Figure 5a,b). The fluorescent dye-filled inside the PDMS cavity diffuses quickly into the surrounding medium, as the cavity offers no diffusion barrier between the medium and the dye-loaded cavity. The diffusion of the fluorescent dye from the hydrogel beads is slow due to the diffusion barrier of the hydrogel polymeric network. The hydrogel beads of the same size as the cavity provide a sustained release of dye to the surrounding medium, and the slow diffusion increases the residence time compared to that of the PDMS cavity. The polymeric network of the hydrogel is shown to offer a barrier to diffusion, reducing the rate of diffusion; the hydrogel embedded into the PDMS reduces the diffusion rate even further because of the unique shape of the cavity. The cavity shape allows the constituents loaded in the hydrogel host to diffuse through the narrow opening, restricting the flow by enclosing most of the surface. The polymeric network and narrow openings greatly reduce drug release, ensuring that the drug remains securely loaded within the contact lenses and minimizing long-term leakage (Figure S10).

The minimum release observed from the hydrogel-embedded PDMS cavity at the completely hydrated state creates an opportunity for a deswelling-based drug release platform. Hydrogels are known to serve a wide variety of applications because of their ability to respond to different stimuli by exhibiting changes in volume. Here, we fabricate a pH-responsive hydrogel that consists of pH-responsive acrylic acid along with the hydrogel precursor. When the surrounding solution pH increased above pH 5, the electrostatic repulsion between the carboxylate ions repelled other acidic pendant groups on the acrylic acid, resulting in swelling of the hydrogel.^28,29^ The swelling ratio and the change in the size of the pH-responsive hydrogels were studied for different pH and found that at lower pH (pH < 6), the swelling is less, and as the pH rises above pH 6, there is significant swelling (Figures 5c and S11). The pH-responsive hydrogels increased swelling at pH 6 and above, in other words, in the ocular pH conditions. The ocular pH for a healthy subject ranges from 6.5 to 7.6.^30^ The tear pH is an important ocular parameter as it is indicative of ocular health, and pH changes are often associated with different ocular conditions like ocular rosacea and pre and postoperative senile cataract patients.^31^ A decrease in tear pH may reveal a corneal infection.^32,33^ The increased swelling ratio or the completely hydrated state of the hydrogel at normal ocular pH levels is suitable to ensure that, at the ocular pH, drug diffusion from the lens is minimal, as the swollen hydrogel inside the PDMS cavity shows only a minimum release. Also, the tear pH can be used as a trigger for the on-demand delivery of a drug depending on ocular conditions. To study the diffusion behavior, pH-responsive hydrogels embedded in the PDMS cavity are hydrated with the fluorescent dye and immersed in the solution, and the fluorescent spectra (FS5 Spectrofluorometer, Edinburgh Instruments) are recorded over time to understand the drug release at different pH levels. The pH-responsive hydrogels were hydrated with the fluorescent dye rhodamine 6G, and the dye diffusion to the solution at pH 5 and 7 was studied by immersing the substrates into the pH solutions and recording the fluorescence spectrum of the solution at regular intervals. The mass released was calculated from the calibration curves obtained using a series of dye solutions (Figure S12). Similar to the previous study, the diffusion to the medium is minimum in the swollen state (pH 7), due to the diffusion barrier offered by the cavity shape and polymeric network. However, the diffusion of the dye to the lower pH medium (pH 5) is significantly high, since the hydrogel deswells substantially at a lower pH (Figure 5d). The deswelling resulting from the change in pH shrinks the hydrogel, pushing the dye out of the polymeric network and to the cavity from which diffusion is quicker. The negligible release from the hydrogel-embedded PDMS cavity ensures that the drug is loaded into these contact lenses, preventing undesired drug leakage during packaging and transportation.

## Conclusions

This study reports the development of a novel contact lens device with hydrogel-embedded microcavity for on-demand drug delivery. The spontaneous self-formation of spherical cavities with high-surface tension liquid droplets in liquid PDMS is used for the fabrication of microcavities with narrow openings of various sizes ranging from a few hundred micrometers to a few millimeters. A method for fabricating smaller cavities by diluting with volatile components, as well as hydrogel-embedded microcavity in PDMS, is demonstrated here. The dilution method aids in fabricating smaller cavities; for a 90% diluted test liquid, the cavity radius is reduced by a factor of nearly 51%. The potential of microcavity-embedded PDMS-based contact lenses for drug delivery is explored. The minimal diffusion of constituents from the swollen hydrogel inside the PDMS microcavity to the surroundings makes it an ideal platform for an on-demand drug delivery platform. The droplet dispensing followed by the thermal curing process allows the fabrication of pH-responsive hydrogel-embedded microcavity on the PDMS substrate. The pH-responsive hydrogel fabricated showed a rapid decrease in swelling at lower pH (pH < 6) and remained more hydrated in ocular pH and above (pH ≥ 6). The pH-responsive swelling property of the hydrogel allows the drug diffusion to be triggered by the change in ocular pH. The tear pH change associated with ocular conditions such as corneal infection could trigger the deswelling of the hydrogel embedded and release the drugs into the ocular environment. The pH-dependent release profile demonstrated that the diffusion process is accelerated at lower pH (pH < 6), and minimal release in higher or ocular pH ensures the on-demand release of the drug. With further development, the potential of this method for on-demand drug delivery can be extended to respond to different ocular stimuli by using various responsive hydrogels. Hence, this novel design can potentially provide dynamic and adaptive drug delivery, triggered by changes in the ocular environment and without human intervention, thereby enhancing bioavailability, prolonging drug residence time, and offering controlled, on-demand release with the potential for multidrug delivery.

## Supporting Information Available

Figure S1: experimental setup consisting of a syringe pump mounted on an XYZ motorized programmable translation stage; Figure S2: microscopic image of the side view of the cavity formed by droplets of different volumes (Scale bar = 400 μm); Figure S3: microscopic image of cavity formed by a droplet diluted with different amount of ethanol (Scale bar = 400 μm); Figure S4: microscopic image of cavity orifice formed by droplet of different volume; Figure S5: variation of cavity radius with the flow rate of the droplet dispensing unit with constant translational stage speed; Figure S6: fabrication schematic of microcavities on PDMS-based contact lens; Figure S7: microscopic image of hydrogel beads before and after swelling with different solvent amounts; Figure S8: microscopic image of hydrogel beads before and after swelling with different volumes; Figure S9: changes in the (b) radius and (c) swelling ratio of HEMA-hydrogel with changing droplet volume; Figure S10: long-term dye release profile from hydrogel-embedded PDMS cavity; Figure S11: change in swelling of the pH-responsive hydrogel at different pH solutions; and Figure S12: calibration curve for Rhodamine 6G fluorescence (PDF)
