# Supplementary material for: Hydrogel-Embedded Polydimethylsiloxane Contact Lens for Ocular Drug Delivery
Source: ACS Appl Bio Mater. 2024 Oct 19;7(11):7324–31. doi: 10.1021/acsabm.4c00975 (PMC11577423; doi:10.1021/acsabm.4c00975)
Supplement: Supplementary file 1 — mt4c00975_si_001.pdf [file mt4c00975_si_001.pdf]

Supporting Information

Hydrogel-Embedded Polydimethylsiloxane Contact Lens for Ocular Drug Delivery

*Aravind Manjeri[1], Sajan Daniel George [1,2 *]*

Corresponding Author Email: *sajan.george@manipal.edu*

[1]Department of Atomic and Molecular Physics, Manipal Academy of Higher Education, Manipal, India – 576104.

[2]Centre for Applied Nanosciences (CAN), Manipal Academy of Higher Education, Manipal, India – 576104.

Contents

Figure S1: The experimental setup consisting of a syringe pump mounted on an XYZ motorized programmable translation stage.

Figure S2: The microscopic image of the side view of the cavity formed by droplets of different volumes (Scale bar= 400 µm).

Figure S3: The microscopic image of cavity formed by a droplet diluted with different amount of ethanol (Scale bar= 400 µm).

Figure S4: The microscopic image of cavity orifice formed by droplet of different volume.

Figure S5: The variation of cavity radius with the flow rate of the droplet dispensing unit with constant translational stage speed.

Figure S6: The fabrication schematic of microcavities on PDMS-based contact lens.

Figure S7: The microscopic image of hydrogel beads before and after swelling with different solvent amounts.

Figure S8: The microscopic image of hydrogel beads before and after swelling with different volumes.

Figure S9: The changes in the b) radius and c) swelling ratio of HEMA-hydrogel with changing droplet volume.

Figure S10: The long term dye release profile from hydrogel embedded PDMS cavity.

Figure S11: The change in swelling of the pH-responsive hydrogel at different pH solutions.

Figure S12: The calibration curve for Rhodamine 6G fluorescence.

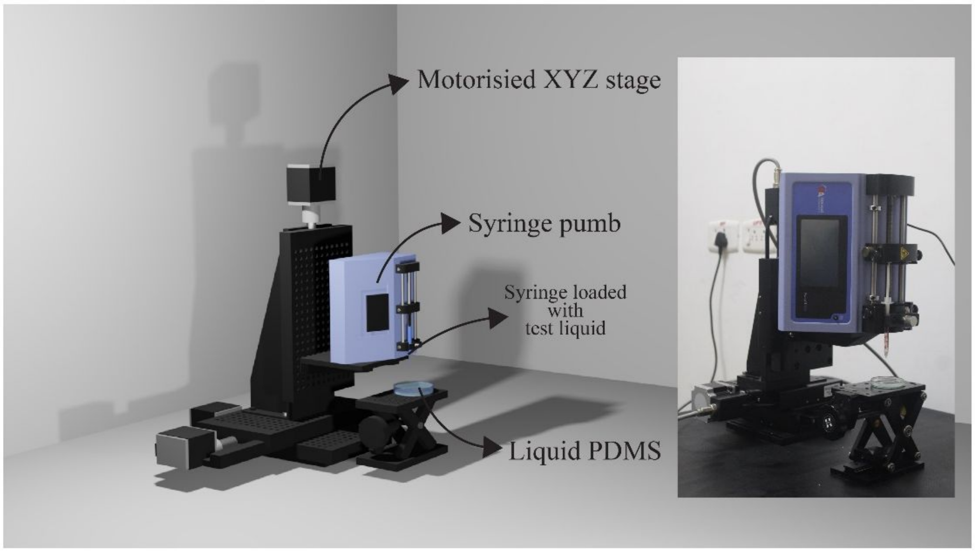

Figure S1: The experimental setup consisting of a syringe pump mounted on an XYZ motorized programmable translation stage.

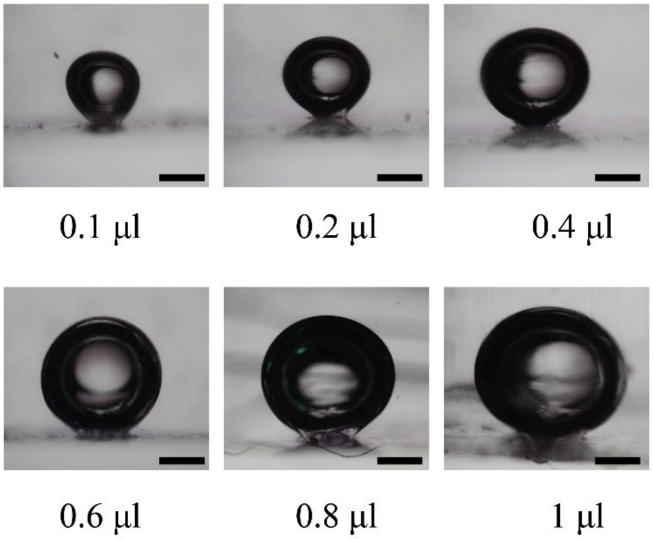

Figure S2: The microscopic image of the side view of the cavity formed by droplets of different volumes (Scale bar= 400 μm).

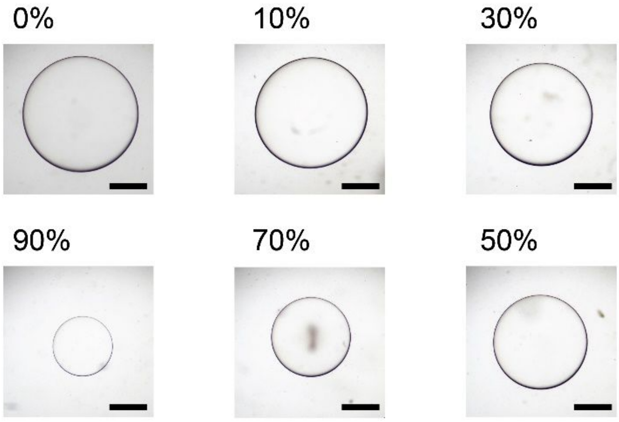

Figure S3: The microscopic image of cavity formed by a droplet diluted with different amount of ethanol (Scale bar= 400 μm).

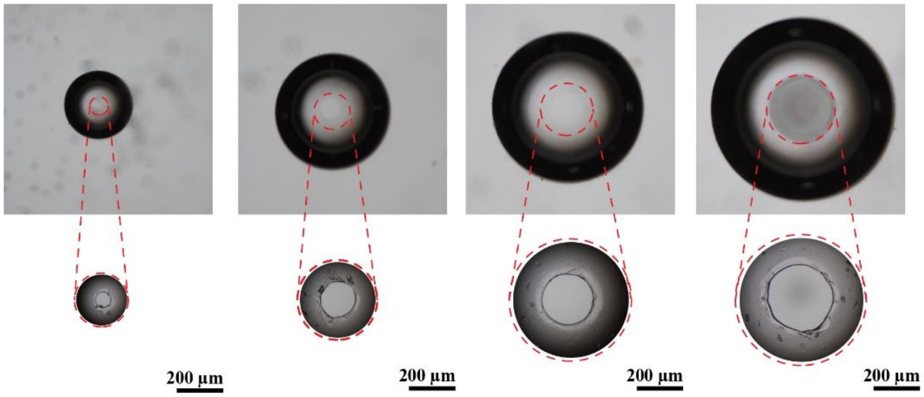

Figure S4: The microscopic image of cavity orifice formed by droplet of different volume.

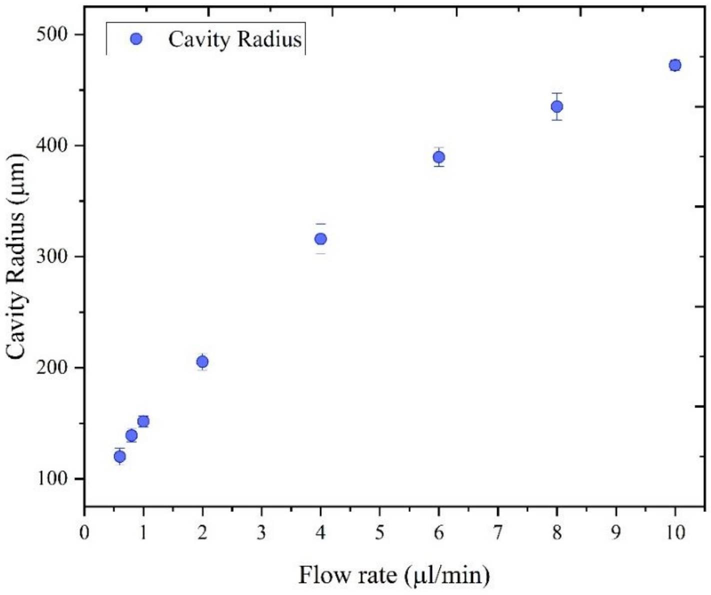

Figure S5: The variation of cavity radius with the flow rate of the droplet dispensing unit with constant translational stage speed.

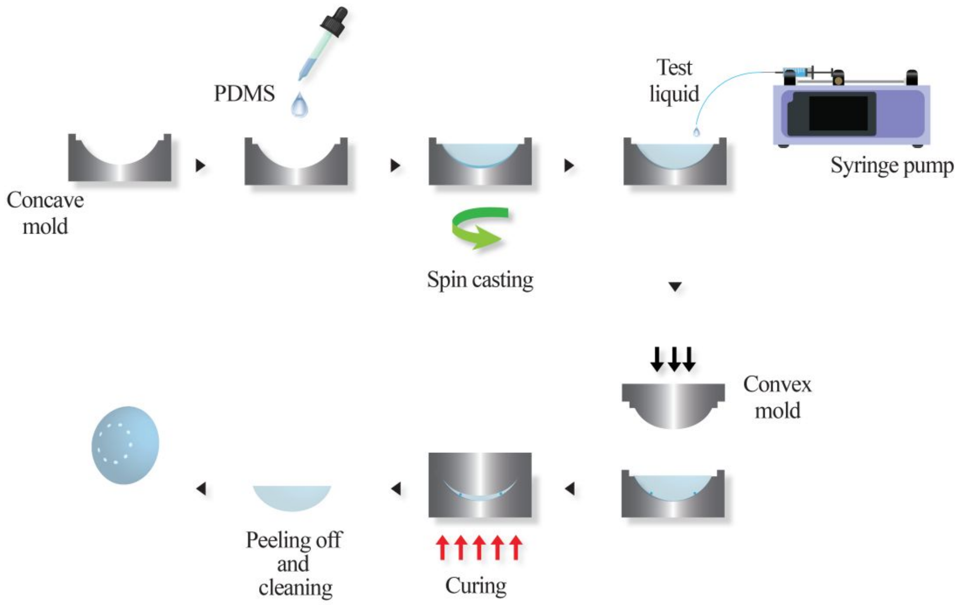

Figure S6: The fabrication schematic of microcavities on PDMS-based contact lens.

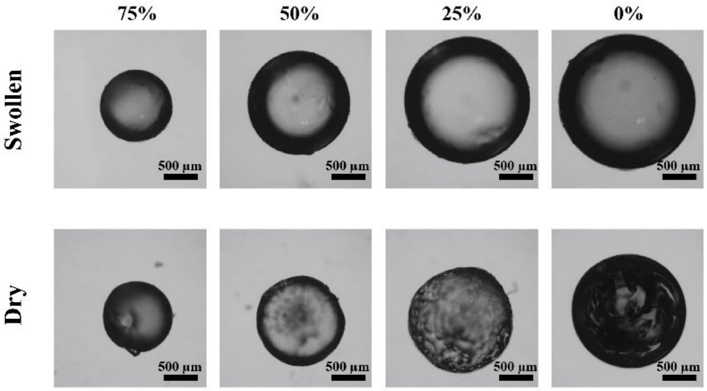

Figure S7: The microscopic image of hydrogel beads before and after swelling with different solvent amounts.

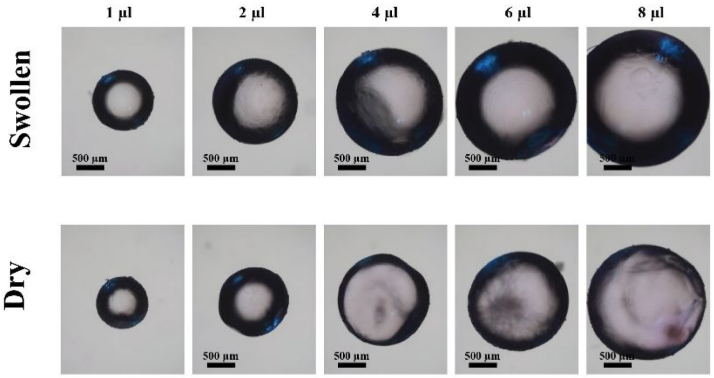

Figure S8: The microscopic image of hydrogel beads before and after swelling with different volumes.

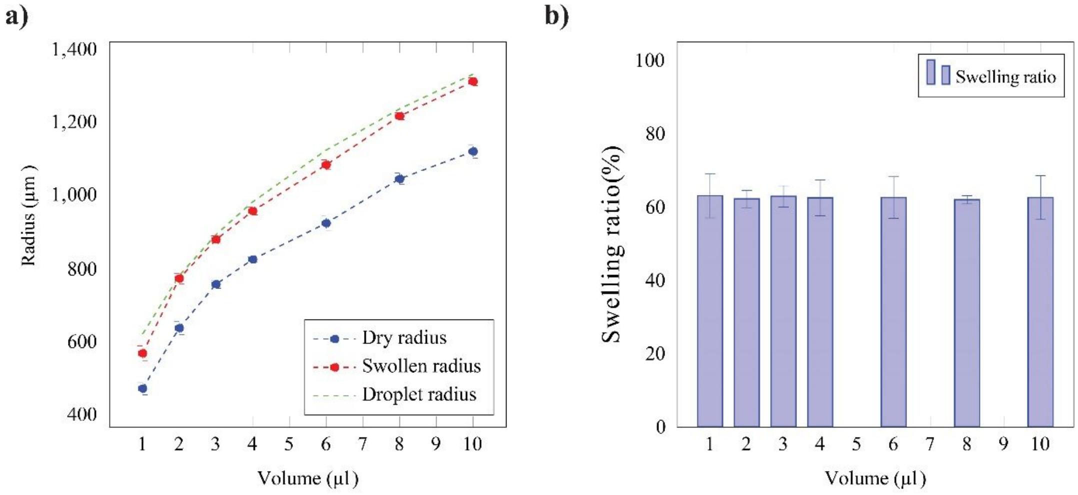

Figure S9: The changes in the a) radius and b) swelling ratio of HEMA-hydrogel with changing droplet volume.

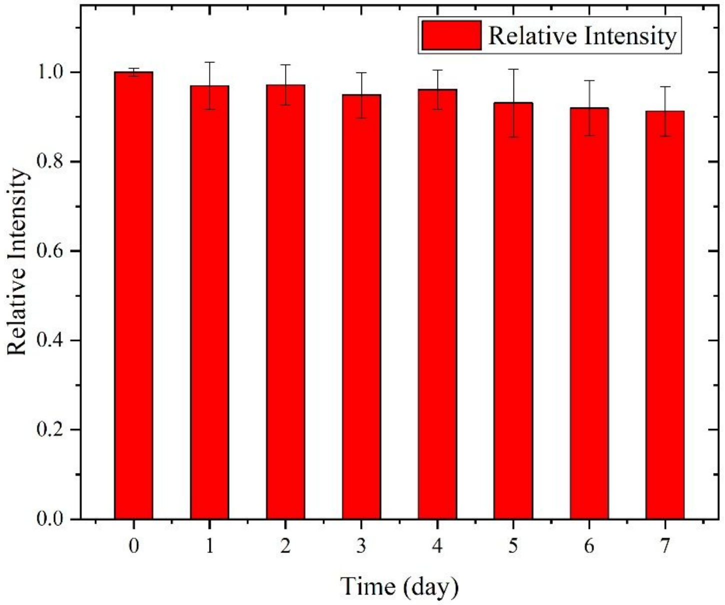

Figure S10: The long term dye release profile from hydrogel embedded PDMS cavity.

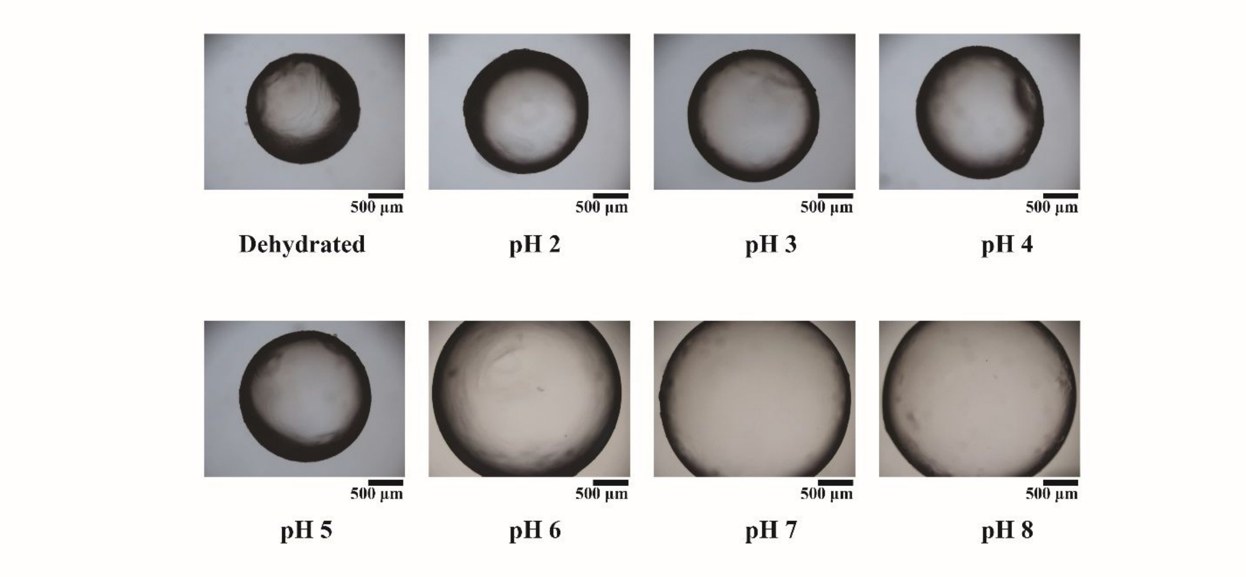

Figure S11: The change in swelling of the pH-responsive hydrogel at different pH solutions.

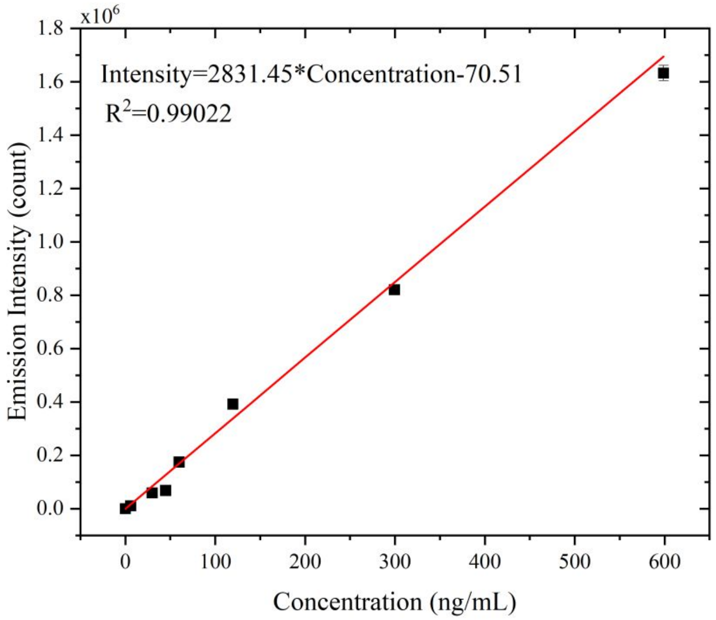

Figure S12: The calibration curve for Rhodamine 6G fluorescence.
